# Tumor Microenvironment: A Metabolic Player that Shapes the Immune Response

**DOI:** 10.3390/ijms21010157

**Published:** 2019-12-25

**Authors:** Shamir Cassim, Jacques Pouyssegur

**Affiliations:** 1 Department of Medical Biology, Centre Scientifique de Monaco, CSM, 98000 Monaco, Monaco; shamir_cassim@yahoo.fr; 2 University Côte d’Azur, IRCAN, CNRS, Centre A. Lacassagne, 06189 Nice, France

**Keywords:** cancer, microenvironment, nutrients, metabolism, tumor acidity, lactate, immunity

## Abstract

Immune cells survey and patrol throughout the body and sometimes take residence in niche environments with distinct cellular subtypes and nutrients that may fluctuate from those in which they matured. Rooted in immune cell physiology are metabolic pathways and metabolites that not only deliver substrates and energy for growth and survival, but also instruct effector functions and cell differentiation. Unlike cancer cells, immune cells are not subject to a “Darwinian evolutionary pressure” that would allow them to adapt to developing tumors but are often irrevocably affected to local nutrient deprivation. Thus, immune cells must metabolically adapt to these changing conditions in order to perform their necessary functions. On the other hand, there is now a growing appreciation that metabolic changes occurring in cancer cells can impact on immune cell functionality and contribute to tumor immune evasion, and as such, there is a considerable and growing interest in developing techniques that target metabolism for immunotherapy. In this review, we discuss the metabolic plasticity displayed by innate and adaptive immune cells and highlight how tumor-derived lactate and tumor acidity restrict immunity. To our knowledge, this review outlines the most recent insights on how tumor microenvironment metabolically instructs immune responsiveness.

## 1. Introduction

Cancers evolve by multiple genetic/epigenetic processes of clonal selection, expansion, within the adaptive landscapes of tissue ecosystems [1]. For several decades, neoplastic cells revealed their capacity to exploit, hijack, and disrupt cellular programs that regulate cell division, survival, and growth, leading to tumor formation and dissemination. The best-known causes of malignant transformation are the genetic and epigenetic modifications that induce stem-cell-like properties, such as unlimited cell division and blocked differentiation [2,3,4]. Metabolism and bioenergetics are central to satisfy the multiple nutrient needs for anabolism and biomass production of malignant proliferating cells [5,6,7,8]. In this context, fermentative glycolysis or “*Warburg effect*”, although low in ATP yield/glucose molecule, represents the best fit for production of anabolic precursors required by rapidly dividing embryonic tissues and tumors [9,10]. However, it becomes now evident that cellular metabolism actively regulates tumorigenicity. For example, loss of the p53 tumor suppressor may be involved in tumor transformation (independently of its well-established functions in DNA repair and senescence), especially through the induction of anabolic pathways including glycolysis, leading then to an early-onset metabolic tumor transformation [11]. Another example of a key role of a mutation-driven metabolic rewiring that favors tumorigenicity is oncometabolites [12]. For example, in human cancers, a consequence of gain-of-function mutations in isocitrate dehydrogenases (IDHs) confers to the enzyme the ability to augment the production of D-2-hydroxyglutarate (D-2HG), an oncometabolite interfering with various α-KG (α-ketoglutarate)-mediated processes, ultimately leading to the inhibition of mitochondrial ATP synthase and activation of a series of downstream signals that involve mammalian target of rapamycin (mTOR) suppression [13,14]. The high glycolytic flux compensates the low ATP yield by a rapid ATP formation and the synthesis of anabolic precursors, nucleotides, amino acids, and lipids. It also induces, in rapidly growing tumors, hypoxic areas with low glucose, and nutrients, and a unique acidic milieu with high lactate concentrations [10,15,16,17]. Importantly, observations from murine in vitro and in vivo models indicate that microenvironmental depletion of glucose and accumulation of lactic acid can have harmful effects on the functionality of the immune cells that were poised to infiltrate and eradicate tumors [15,18,19,20].

Cancers are highly heterogeneous, and a broad spectrum of immune cells can infiltrate human tumor tissues [21]. Among adaptive immune cells, the tumor-infiltrating T cells are the best documented. Various phenotypic sub-populations (CD4^+^ and CD8^+^), functional (effector, memory), and differentiation (CD4^+^ T helper 1 (Th1), CD4^+^ T helper 17 (Th17), CD4^+^ Treg) states of T cells have been described [22,23,24]. T cells can impact on tumor growth either through direct engagement or through stimulation of other cells found in the tumor microenvironment. Notably, this feature has been used in clinical settings that aim to enhance their anti-tumor effect, including T-cell-inhibitory PD-1 receptor blockade or by ex vivo engineered chimeric antigen receptor (CAR)-transduced T cells [25].

The interaction of innate and adaptive immune cells is fundamental for an effective response. The first immune cells found in human tumors were innate cells and more specifically macrophages [26]. Although their normal role in physiological conditions is in promoting both innate and adaptive immunity (phagocytosis of dead or dying cells and cell debris), tumors have largely reeducated them to a phenotype that promotes tumor growth and spread [27]. Macrophages can polarize toward an anti-inflammatory phenotype with pro-tumoral properties through alternative activation (M2) when stimulated with IL-4 and IL-10—in contrast to M1 pro-inflammatory macrophages, which display anti-tumor effects [27,28]. M1 and M2 macrophages are key players during inflammation as they modulate tissue homeostasis and repair through these distinct functional specialties [29]. Growing evidence indicates that macrophages use distinct metabolic pathways during M1 and M2 activation: M1 macrophages boost their anabolic metabolism (anaerobic glycolysis, pentose phosphate pathway, and fatty acid biosynthesis), whereas M2 macrophages favor catabolic metabolism and primarily rely on oxidative phosphorylation (OXPHOS) to sustain their metabolic requirements [30]. These characteristics provide interesting metabolic checkpoints to fine-tune macrophage deleterious behavior in diseases, especially in the tumor microenvironment.

How metabolism regulates immune cells differentiation, function, plasticity, and how their intracellular metabolism can affect their functionality is currently a very hot topic. As previously introduced, tumor progression is characterized by a tangled network of relationships among different cell types that collectively exploit metabolic rewiring and mutually influence their functionality [19,31]. Although the development of several monoclonal antibody-based therapies has shown unprecedented responses in some cancer patients, the response rates still remain low and transient [32,33]. These observations could potentially stem from multiple mechanisms suppressing anti-tumor immune functions within an unfavorable tumor milieu and metabolism. 

## 2. Tumor Cell Metabolic Features Impacts on Local Nutrients that are Critical for Immune Cell Function

### 2.1. Glucose Metabolism

Maintenance of cellular bioenergetics is essential for all living cells, including immune cells and more particularly lymphocytes [34]. As T effector cells have to move from a nutrient-replete environment in the lymph node or spleen to distant sites of infection, they are likely to experience more restrictive metabolic environments. Bioenergetic profiling of T cells has revealed that the metabolism of T cells dynamically changes during activation (antigen encountering) to perform effector functions [35]. T cells switch to a program of anabolic growth and biomass accumulation, which by definition dictates increased demand for ATP and metabolic resources to generate daughter cells. Therefore, a shift of activated T cells toward aerobic glycolysis has been noticed [35,36]. This rewiring is orchestrated by T cell receptor (TCR) signaling, which promotes the coordinated up-regulation of glucose and amino acid transporters to adjust nutrient uptake and facilitate T cell blastogenesis. In parallel, catabolic pathways generating ATP such as fatty acid β-oxidation (FAO) are actively suppressed [37]. Consistent with the metabolism of other non-proliferating cells, resting naïve T cells (that have not yet encountered antigen) sustain, however, lower rates of glycolysis and predominantly oxidize glucose-derived pyruvate via OXPHOS or engage FAO to produce ATP [31]. Similarly, long-lived antigen-specific memory T cells are considered as a quiescent population. They adopt a metabolic profile similar to that of naïve T cells: an increased reliance on OXPHOS and lower rates of nutrient uptake and biosynthesis, in agreement with their increase in mitochondrial mass [31]. A recent report demonstrated that an enhanced glycolytic metabolism of neonatal CD8^+^ T cells was sufficient to abrogate the formation of memory CD8^+^ T cells [38]. Accordingly, this mitochondrial reliance has shown to provide a bioenergetic advantage during secondary exposure to antigen through the rapid mitochondrial ATP production upon TCR engagement [39]. Memory T cells may then be perceived as being metabolically primed, with mitochondrial metabolism fueling the rapid recall response to reinfection.

**Figure 1:**
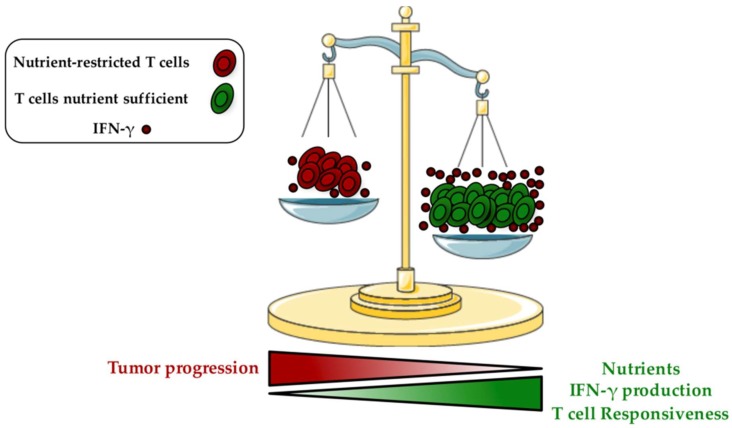
Metabolic competition in the tumor microenvironment is a driver of T cell responsiveness. As mentioned in the review’s text, the increased metabolism displayed by tumor cells consumes most nutrients from the surrounding microenvironment, and as a consequence, impacts on the intrinsic metabolism of T cells, with a decreased ability to produce cytokines (especially IFN-γ) and to develop into tumor-specific T effector cells, leading to T cell hypo-responsiveness and increased tumor progression (nutrient-restricted T cells in red). Conversely, in a nutrient-enriched microenvironment, T cell metabolism increases (glycolysis) and leads to an improved IFN-γ production and immune response (T cells nutrient sufficient in green). IFN-γ: Interferon gamma.

Emerging evidences suggest: (1) that the metabolic alterations of T cells are critical to impair anti-tumor immunity, and (2) that neoplastic cells are the most important players mediating this immune suppression [40,41]. In fact, metabolic interplay and nutrient competition between cancer cells and T cells exist and are recognized as key drivers of carcinogenesis. The increased glucose addiction and glycolysis rate of rapidly growing cancer cells (Warburg effect) consume most nutrients from the surrounding microenvironment [15,42,43]. As a consequence, the tumor-imposed metabolic restrictions dramatically reduce T cell responsiveness. Importantly, a down-regulation of the glycolytic machinery has been detected and these T cells became unable to produce cytokines and to develop into tumor-specific T effector cells, leading to a state of anergy [44]. Glucose deprivation can prevent tumor infiltrating CD8^+^ T cells function by altering interferon gamma (IFN-γ) production, a key effector molecule having pro-inflammatory and enhanced anti-tumor properties [45] (Figure 1). It has been proposed that these effects are mediated through the glycolytic glyceraldehyde-3-phosphate dehydrogenase (GAPDH) enzyme, by preventing translation of IFN-γ under low glycolytic flux [45]. AMP-activated protein kinase (AMPK), which is activated under poor nutrient conditions by an increase of AMP:ATP ratio, also plays a key role in regulating Ifng mRNA translation [46]. Recent findings demonstrated that the selective deletion of AMPK in T cells hampers IFN-γ and Granzyme B production in intratumoral CD8+ T cells [47]. The absence of glucose can also suppress T cell receptor (TCR)-dependent activation of Ca^2+^ and nuclear factor of activated T cells (NFAT) signaling through phosphoenolpyruvate, which maintains Ca^2+^ and NFAT by blocking sarco/endoplasmic reticulum Ca2+-ATPase, and leading then to T cell hypo-responsiveness [48].

CD4^+^ Treg cells hamper inflammation and are (1) often associated with increased tumorigenicity, and (2) related to poor prognosis when detected in solid tumors of patients with cancer [49]. Indeed, tumor infiltrating Treg cells were shown to restrict local anti-tumor immunity. As a main contrast to T effector cells that suffer from the tumor microenvironment to sustain functionality, Tregs feel comfortable [50]. One possible explanation could stem from the modulation of their sensitive metabolic pathways. As reported in murine models, Treg cells express low levels of GLUT-1 and do not depend on glucose uptake and glycolysis [51]. Similar to non-proliferating memory T cells, they prefer to rely on OXPHOS and lipid oxidation to favor ATP production. Forkhead box protein P3 (FOXP3), the lineage-defining transcription factor of murine Treg cells, was proposed to be a key regulator of this phenotype [52]. Mechanistically, FOXP3 can induce the expression of genes involved in lipid metabolism and down-regulate genes involved in glucose uptake and glycolysis. Importantly, the PI3K/AKT/mTORC1 axis, a major player in the induction of glycolysis, was abolished when expression of FOXP3 was forced [52]. Unexpectedly, glucose abundancy may be important for Treg induction, as glycolysis in conventional CD4^+^ T cells is crucial for the initiation of the regulatory phenotype via the translocation of Enolase-1 glycolytic enzyme to the nucleus, where it can bind to FOXP3 regulatory loci [53].

B lymphocytes provide adaptive immunity by generating antigen-specific antibodies and by supporting the activation of T cells. B cells are highly metabolically active, but little is known about how global metabolism supports their activation. Similarly to what occurs upon T cell activation, an increase of glucose uptake and lactate production has been evidenced in naïve B cells after stimulation [54,55]. Also, a marked augmentation of glycolytic activity of germinal center B cells was described [54]. Surprisingly, a recent study from Waters et al. revealed an unexpected role of tricarboxylic acid (TCA) and OXPHOS in the activation of naïve B cells [56]. Indeed, although activated B cells have been shown to increase glucose uptake, glucose deprivation did not show to impair neither their growth nor functionality [56]. However, glutamine restriction or inhibition of OXPHOS with 10 nM Oligomycin impaired B cell growth and differentiation [56]. This discrepancy is most likely due to the fact that the augmentation of extracellular acidification observed upon B cell activation was not reflective of any increase of extracellular lactate, but rather to the enhanced CO_2_ and carbonic acid production arising from a highly active TCA cycle [56,57,58]. We speculate this nutrient competition between cancer cells and adaptive immune B cells is crucial in the formation of an immunosuppressive milieu.

Natural killer (NK) cells are important anti-cancer effector cells. They have excellent potential for immunotherapy although impaired functions during cancer limit their effectiveness. Upon activation, NK cells increase aerobic glycolysis [59]. With high IL-15 stimulation, NK cells elevate the activity of mTOR to favor bioenergetic metabolism, increase glucose uptake, and up-regulate the expression of transferrin receptor CD71 and amino acid transporter chaperon CD98 [60]. This process was shown to be essential for sustaining NK cell proliferation during development and the acquisition of cytolytic potential. Accordingly, impairment of glucose metabolism and disruption of mTOR signaling leads to a diminished cytotoxic activity in NK cells [61]. A recent report revealed that sterol regulatory element- binding protein (Srebp) transcription factors play an important role in the cytokine-induced metabolic reprogramming of NK cells by increasing both glycolysis and OXPHOS [62]. Furthermore, Srebp inhibition prevented this phenotype and decreased NK cell cytotoxicity [62]. However, it remains unclear whether metabolic alterations found in tumors may affect the metabolic activity and the Srebp-mediated NK cell function.

Neutrophils are a vital component of immune protection. However, in cancer, they promote tumor progression by increasing invasion and metastasis through releasing proteases, increasing angiogenesis, and directly promoting tumor growth [63,64,65,66,67]. Furthermore, neutrophils have been shown to limit anti-tumor immune responses by suppressing T cell and NK cell activity, partly by generating reactive oxygen species (ROS) that disrupts lymphocyte functions [68,69]. Traditionally, neutrophils have been thought to be a highly glycolytic population, dependent on glucose, with little or no mitochondrial function except to drive apoptosis [70]. However, neutrophil metabolism has recently gained interest as the importance of mitochondria in effector functions such as chemotaxis and the generation of neutrophil extracellular traps (NETs) have come to light [71,72]. A recent report showed that cancer-associated neutrophils employ their mitochondrial respiratory capacity to support the generation of ROS in conditions where glucose utilization, and therefore pentose phosphate pathway (PPP) derived NADPH, is limited [73]. These data suggest that oxidative neutrophils benefit tumor growth as, unlike glycolytic-neutrophils from a healthy host, they can maintain ROS-mediated suppression of T cells under nutrient restricted conditions, such as the low glucose environment of advanced tumors. These results emphasize the promising role that neutrophil mitochondrial metabolism may have for cancer therapy, and inexorably underlines the competition for fuels shared by tumor cells and immune cells of the microenvironment.

### 2.2. Amino Acid Metabolism

Glutamine, a nonessential amino acid, is the most abundant nutrient in the blood and constitutes an essential substrate for T cells activation and growth process. When T cells are activated through efficient TCR signaling, the uptake and biosynthesis of amino acids are widely increased [74,75]. Glutamine catabolism is intensely induced in active T cells supplying intermediate metabolites required for different pathways of biosynthesis and substrates for mitochondria [76,77]. During glutaminolysis, glutamine carbon backbone can be converted (1) to glutamate favoring cystine import via the xCT antiporter, (2) to α-KG to maintain TCA cycle homeostasis, or (3) to lactate that generates NAD and NADPH [78,79]. During T cell activation, glutamine can be used, providing pyruvates to overcome intense aerobic glycolysis levels [46]. T cell glutamine uptake depends on the neutral amino acid transporter type 2 (ASCT2) and its genetic ablation has been shown to prevent the induction of Th1 and Th17 cells [80]. In line with this, glutamine deprivation supported the differentiation into Tregs and addition of α-KG reversed this effect and rescued Th1 differentiation under glutamine deprivation through the induction of Tbet, a T effector cell transcription factor, which correlated with increased mTORC1 signaling [81]. Moreover, 6-diazo-5-oxo-L-norleucine, a naturally occurring antagonist of glutamine, inhibited glutamine metabolism in activated T cells and was able to inhibit immune-mediated rejection of allografts in fully mismatched skin and heart allograft transplantation models [82]. Similarly, glutamine was reported to be essential for B-cell proliferation and differentiation into plasma cells [83]. Since many cancer types harbor mutated MYC, which transcriptionally induces mitochondrial glutaminolysis and leads to glutamine addiction of cancer cells, we speculate that glutamine could become a limiting metabolite that may have a pivotal role in tumor-induced immunosuppression [84].

Tryptophan and arginine have also been proposed to be critical for T cell activation and function. This concept has gained interest because of the tumor-induced extracellular depletion of these amino acids, thereby altering T cell activity, and causing their anergy. Tryptophan is an essential amino acid required for the production of several important molecules and its catabolism through the kynurenine pathway generate metabolites such as kynurenine, kynurenic acid, 3-hydroxy-kynurenine, and 3-hydroxy-anthranilic acid [85]. Several studies indicated that tryptophan plays a key role in T cell survival and activation whereas its metabolites (1) eliminate T cell function and (2) are able to induce T cell apoptosis [86]. In parallel, neoplastic cells often overexpress the amino-acid-catabolic enzyme indolamine-2,3-dioxygenase (IDO), which can lead to extracellular depletion of tryptophan [87]. T effector cells then become affected by the local diminution in tryptophan concentrations and decrease their functionality [86]. Mechanistically, tryptophan depletion activates general control nonderepressible 2 (GCN2), a stress-response kinase that is activated by elevations in uncharged transfer RNA (tRNA), leading to inhibition of T cell function, impaired Th17 differentiation and promotion of Treg development [88,89]. Three enzymes have been recognized in modulating tryptophan degradation through the kynurenine pathway: (1) tryptophan-2,3-dioxygenase, (2) indoleamine 2,3-dioxygenase 1, and (3) indoleamine 2,3-dioxygenase. Thus, tryptophan degradation remains one of the resistance mechanisms adopted by tumors to avoid immune suppression, and in an hostile tumor microenvironment context, such inhibition results in the suppression of anti-tumor immune responses [90,91,92].

Arginine was also revealed as a central amino acid in the function of T cells. This multifunctional amino acid is involved in protein synthesis and in generating several metabolites precursors including polyamines and nitric oxide involved in immunometabolism [93]. The absence of extracellular arginine or enzymes responsible of de novo synthesizing arginine (Argininosuccinate 1 (ASS1)) has been found to impair T cell proliferation, aerobic glycolysis, and reduce cytokine production and expression of activation markers such as CD25 and CD28 [94,95]. Importantly, deletion of ASS1 was shown to prevent in vitro Th1 and Th17 cell polarization, even in the presence of extracellular arginine [96]. Further, a recent report indicated that increased arginine levels promote survival capacity of T memory cells and anti-tumor activity in an OVA-antigen-expressing B16 melanoma mouse model [93]. According to the beneficial effects of arginine and tryptophan on T cell metabolic adaptation and anti-tumor activity, both amino acids would be exploited as an attractive target for therapeutic intervention in anti-tumor response [97,98].

Cysteine amino acid is widely used throughout the cell for diverse roles including catalysis, protein folding, trafficking, and mediating the major antioxidant defense [99,100]. Although protein synthesis accounts for the majority of cellular cysteine usage, another essential use of cysteine is the production of the tripeptide glutathione (GSH) for antioxidant defense and maintenance of thiol status [99,101]. Cysteine can be easily oxidized to form a dimer containing disulfide bridge between two cysteines called cystine, and both are transported over the plasma membrane by ASCT1, ASCT2 (although controversial), and by xCT (which together with CD98/Slc3a2 form system x_c_^−^ cystine/glutamate antiporter), respectively [102,103]. Although it has been reported that T cells require GSH for proliferation both in vitro and in vivo [104,105], previous studies have been conflicting as to whether T cells accumulate cysteine indirectly via uptake of cysteine secreted by antigen presenting cells (APCs) or directly through xCT-mediated import of cystine [106,107]. Since prior results indicated that T cells express ASCT1/ASCT2 transporters but not xCT, it was first proposed that a sufficient high concentration of exogenous cysteine is provided to T cells by APCs, and that this dependency is needed for T cell activation [108,109]. However, other studies showed (1) that purified T cells can be fully activated in the absence of APC [104,110], and (2) that xCT expression can be induced following T cell activation [111]. In line with these observations, Levring et al. confirmed that while naïve T cells express very low levels of both cysteine and cystine transporters, activated T lymphocytes display a strong up-regulation of these transporters, thus enabling T cell responsiveness independently of APC-released cysteine [106]. These results support a T cell-autonomous requirement for ASCT1, ASCT2, and xCT functions in cultured lymphocytes. Interestingly however, Arensmani et al. recently showed that T cell-specific knockout of xCT does not disrupt the anti-tumor T cell response in vivo [112]. This inherently different requirements for xCT in vitro versus in vivo may stem from the well-established discrepancies between the tissue culture environment and the physiologic niches where T cells respond to antigenic stimuli. Indeed, (1) T lymphocytes are exposed to much higher levels of oxygen under standard tissue culture conditions than they experience in peripheral tissues [113], and (2) cysteine is present at different levels in culture medium (where it is rapidly oxidized to cystine) in comparison to in vivo peripheral tissues [7]. Thus, although cystine levels surpass those of cysteine found in plasma, cysteine remains, however, present at low concentrations in blood, and the probability that circulating cysteine is enough to support T cell proliferation in vivo cannot be rejected. In the same vein, a growing body of evidence also revealed that cysteine is critical for cancer cell proliferation and survival [114,115]. The metabolic demands placed upon a tumor cell produce unique needs that must be met through extracellular sources of cysteine [116]. In turn, when extracellular cysteine levels decrease and become limiting, endogenous transsulfuration activity can support in vivo cancer cell growth and proliferation through the generation of de novo cysteine [117]. Moreover, inhibiting transsulfuration pathway activity of hepatocellular carcinoma (HCC) cells by methylation of cystathionine β-synthase promoter resulted in an increased reliance of these cells to import cystine through xCT [116,118,119,120]. Hence, acquisition of cysteine from extracellular cystine by tumor cells remains a vital strategy to maintain GSH levels and buffer oxidative stress that would otherwise cause cell death [115]. Once again, these last observations highlight the metabolic competition that exists between cancer and immune cells: local cysteine can be metabolically used by tumors to enhance their aggressiveness, and as a result repress T cell effectiveness leading to the suppression of anti-tumor immune responses.

Other limiting amino acids including serine and alanine also revealed their importance in promoting T cell effector functions. Recently, it was reported that extracellular serine is required to support de novo purine biosynthesis of proliferating T cells: when cultured without exogenous serine, T lymphocytes failed to proliferate efficiently in vitro [121]. Moreover, following Listeria infection, mice maintained on a serine-restricted diet also showed a significantly reduced number of IFN-γ-producing CD8^+^/CD4^+^ T cells, indicating that antigen-specific T cell responses were identically affected by serine restriction in vivo [121]. However, the quantity of cytokine produced on a per-cell basis by T effector cells responding to LmOVA (Listeria monocytogenes expressing OVA) was not affected by the restricted diet, suggesting that dietary serine deprivation did not actually affect the functionality of the T cells that could respond to infection, but rather the quantity [121]. Similarly, Ron-Harel et al. recently identified T cell reliance on extracellular alanine for initial activation and protein synthesis [122]. Indeed, activated T cells cultured in alanine-free media displayed diminished effector functions as evidenced by the reduced levels of secreted pro-inflammatory cytokines, such as interleukin-17 (IL-17), IFN-γ, and interleukin-6 (IL-6) [122]. Concomitantly, alanine-labeled fraction (using [U-^15^N^13^C]-alanine) in total cell proteome of activated T cells revealed that alanine deprivation could prevent activation-induced protein synthesis [122]. Since reduced levels of alanine were depicted in some tumors [123], one may suggest that the control of local alanine levels through the uptake or secretion of alanine by resident cells in the lymphatic tissue (or other tissues where resident memory T cells can get re-activated) may impact T cell activation.

### 2.3. Oxygen

A common feature of most rapidly growing tumors is a low level of oxygen called hypoxia. Indeed, in intensively proliferating and expanding tumor tissues, oxygen supply is often limited, by the distance between cells and the existing vasculature creating even more hypoxic milieu [124]. Hypoxia can function as a metabolic adjunct to further promote a malignant phenotype. Indeed, hypoxic tumor cells display enhanced glucose uptake and glycolysis through induction of all glycolytic genes, and elevated glycolysis is associated with sustained malignant growth [16,125,126]. Mechanistically, hypoxia-inducible factor 1 (HIF1) actively suppresses TCA cycle metabolism by directly trans-activating the gene encoding pyruvate dehydrogenase kinase 1 (PDK1), leading then to inactivation of the pyruvate dehydrogenase complex and subsequent loss of pyruvate oxidation [125,127].

The hypoxia-induced effects on immune cell activation have been conflicting as to whether low oxygen tension favor or repress T cell responsiveness. On the one hand, hypoxic conditions lead to less efficient TCR- and CD28-mediated T cell activation [128]. Also, HIF1α-deficient CD4^+^ and CD8^+^ T cells from Lck-Cre/HIF1-floxed mice show an improved capacity to proliferate and to secrete IFN-γ [129]. Conversely, it has been demonstrated that HIF1α does not impact on the proliferation of T cells, but rather support the differentiation of Th17 cells via direct transcriptional induction of the RAR-related orphan receptor gamma (RORγt) [130]. Unexpectedly, HIF1α was also shown to increase the expression of CD137 costimulatory molecule on tumor infiltrating T cells [131].

Oxygen is necessary for OXPHOS and the production of ROS. At a low or moderate concentration, ROS were found to be essential for T cell effectiveness and antigen-specific proliferation [132]. However, a strong impairment in the functionality of immune cells could be evidenced at high levels of ROS due to a down-regulation of the CD3ζ chain [133,134,135]. Considering the paradoxical effect of ROS on T cell effector functions, a tight balance between production and consumption of ROS should be accomplished to potentiate anti-tumor activity.

Macrophages are sensitive to variations in oxygen availability. It has been reported that anti-inflammatory M2 macrophages accumulate in hypoxic tumor regions, whereas the pro-inflammatory M1 macrophages reside in normoxic regions [136,137]. Indeed, M2 macrophages are involved in matrix remodeling, tissue repair, and angiogenesis, and in promoting genetic instability, whereas M1 macrophages display important microbicidal activity and cell proliferation inhibitory capacity [138]. Mechanistically, intratumoral hypoxia-induced semaphorin 3A attracts tumor-associated macrophages (TAMs) to hypoxic regions by triggering vascular endothelial growth factor (VEGF) receptor 1 phosphorylation [136]. Additionally, hypoxic TAMs can up-regulate the expression of REDD1 (regulated in development and DNA damage responses 1), thus inhibiting mTOR activity, and leading to (1) a decrease of glycolysis, (2) abnormal blood vessel formation, and (3) promotion of metastases [139]. Similarly, hypoxic TAMs are able to secrete proteolytic enzymes, such as matrix metalloproteinases 1 and 7, and contribute to cell proliferation and tumor dissemination [140,141]. Importantly, the depiction of macrophages polarization has led scientists to reconsider their concept on how immunity functions, as anti-inflammatory properties were usually shown to prevent tumor growth—in this case, the anti-inflammatory phenotype of M2 macrophages is rather associated with their capacity to repress anti-tumor immune functions, and the M1/M2 polarization should then be considered as a simplified conceptual framework describing a continuum of different functional states [142].

## 3. Metabolic By-Products of Tumor Cells Impact on Immunity

### 3.1. Glucose Metabolism, Lactate, and Tumor Acidity

Although the concentration of essential nutrients may be poorer in the tumor microenvironment when compared to normal tissues, several products of tumor cell metabolism accumulate and thereby affect immune cell function. The most prominent metabolite in the microenvironment of highly glycolytic tumors remains lactate that can reach up to 30–40 mM in some tumor areas [143,144]. Associated with lactate are protons (H^+^), both co-transported out of the cell by the monocarboxylate transporters (MCT1 and 4) [145,146,147]. This leads to an accumulation of lactate and to a decreased pH in the extracellular space. Brand et al. demonstrated that lactate dehydrogenase A (LDHA)-mediated production of lactate in tumor cells and subsequent acidification can: (1) restrict IFN-γ production in tumor infiltrating T cells, and (2) prevent NK cell activation, resulting in a loss of immune surveillance and promoting tumor growth in a mouse melanoma model [20,148]. In connection with this last observation, innate immune cells also showed sensitivity to tumor-generated lactate [149]. Indeed, it has been shown that tumor-derived lactic acid can reduce the differentiation and effector function of monocytes both in vitro and in vivo [148,150]. However, data on the effect of lactate itself on macrophage polarization and function are still under debate. While it has been advocated that lactic acid can augment toll-like receptor (TLR) 4-mediated signaling, nuclear factor (NF)-κB-dependent gene regulation, and the pro-inflammatory function of macrophages [151], other studies have shown opposite effects [143,152,153]. In particular, it has been demonstrated that high concentrations of lactate could also stimulate the polarization of anti-inflammatory M2 macrophages through the stabilization of HIF1α [153] or, as recently reported, by lactylation of histones [154]. Lactic acid can also play a crucial role on the phenotype and functionality of dendritic cells (DCs) with: (1) reduced basal CD1 expression (a major histocompatibility complex (MHC) class 1 molecule triggering the immune response), (2) maintenance of a tolerogenic phenotype characterized by diminished IL-12 and increased IL-10 secretion in response to TLR stimulation, and (3) impaired migratory response to lymph node-derived chemokine [155,156].

**Figure 2:**
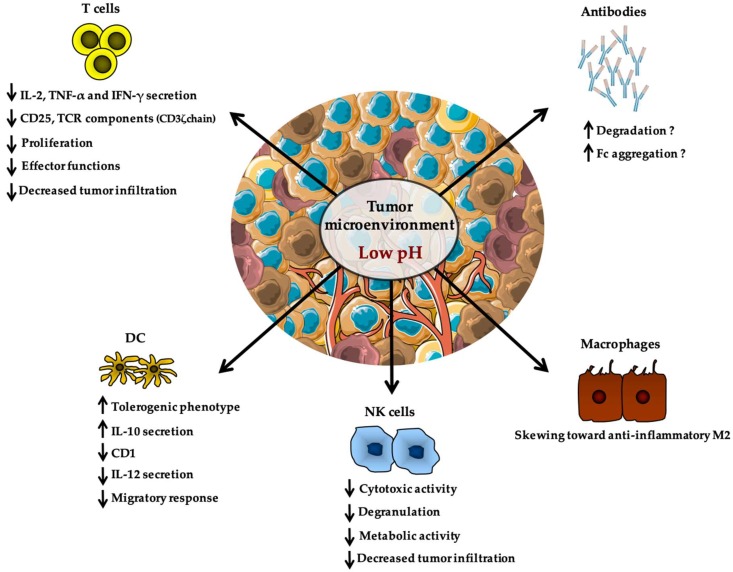
Tumor acidity impacts on immune cell function. As illustrated in the review’s text, tumor acidity acts as an immune escape mechanism by which tumor cells (whose heterogeneity is depicted by tumor cells of different colors) repress the activity of anti-tumor immune effectors (including T cells, natural killer (NK) cells, and dendritic cells (DC)), and also favor the conversion of macrophages toward a non-inflammatory M2 phenotype, potentiated by hypoxia and altered metabolism, thereby creating an hostile milieu for T cells, NK cells, and DC. However, the possible effect of pH on antibody activity is still controversial and not fully elucidated. IL-2: Interleukine 2, TNF-α: Tumor necrosis factor-α, IFN-γ: Interferon gamma, TCR: T cell receptor, IL-12: Interleukine 12, IL-10: Interleukine 10, DC: Dendritic cell, NK: Natural killer, Fc: Fragment crystallizable.

The deleterious impact of lactate on immune cells is often in concert with a decreased pH in the tumor microenvironment and acidity was also reported to have distinct effects on a variety of immune populations (Figure 2). This was first described by Fischer et al. who demonstrated that low extracellular pH leads to decreased cytokine production and to a loss of cytotoxic effector functions without affecting cell viability [157,158]. In 2001, Bosticardo et al. reported that pH as low as 6.6 leads to impaired activation and proliferation of T cells as evidenced by altered expression of the high-affinity IL-2 receptor CD25, as well as diminished cytokine secretion and cell cycle progression [159]. Importantly, providing stronger T cell activation was sufficient to restore complete function, indicating that acidity might raise the activation threshold of T cells [159]. Similarly, a pH of 6.5 resulted in a declined responsiveness of tumor infiltrating T cells from melanoma patients, with decreased expression of TCR components (such as CD3ζ chain) and impaired secretion of IL-2, tumor necrosis factor-α (TNF-α), and IFN-γ [160]. Consistent with these findings, high-dose administration of esomeprazole (a proton pump inhibitor largely used in clinical setting for indigestion and gastric protection) was associated with an increase of tumor pH, paralleled by a boost of T cell infiltration and anergy reversion that could be selectively detected at tumor site of melanoma-bearing mice but not in tumor-free organs [160]. Accordingly, a recent report showed that neutralization of tumor acidity with bicarbonate therapy increased T cell infiltration and impaired tumor growth [161]. Tumor-derived acidity also affects cells of innate immunity as evidenced by the enhanced endocytosis capacity of DCs when cultured at a pH of 6.5 [149,162] (Figure 2). DCs pulsed with antigens at low pH values also displayed an improved efficacy in inducing specific cytotoxic responses mediated by CD8^+^ T cells as well as specific antibody responses in vivo [162]. Also, a role for acidic pH in regulating macrophage polarization recently appeared and showed to be mediated by G protein-coupled receptors (GPCRs) and cyclic AMP production [163,164] (Figure 2). Indeed, activation of GPCR signaling induced by tumor acidosis has been shown to increase expression of the transcription factor inducible cyclic AMP early repressor (ICER), which in turn stimulated polarization of macrophages toward a non-inflammatory M2 phenotype, and thus promoted in vivo tumor growth [164]. Importantly, mice with myeloid-specific deficiency of ICER could resist the growth of highly glycolytic tumors [164]. Finally, along the line of this section, it was reported that restricted glycolysis and acidosis of mouse melanoma preserves T cell effector functions and augment checkpoint therapy [165].

The possible effect of pH on antibody activity is still conflicting and no consensus on a unique effect of these effectors in tumor immunosurveillance has been yielded [166] (Figure 2). Since monoclonal antibodies (mAbs) represent a new class of therapeutic drugs broadly used for the treatment of many solid tumors, understanding whether tumor acidity might influence their functionality could provide novel insights leading to improved clinical efficacy of cancer treatments [167,168,169]. However, no direct study addressing this topic is, to our knowledge, available in literature. For this reason, we prefer not to go into more details, even if, based on the molecular and structural features of mAbs and their biodistribution properties in the tumor microenvironment, some interesting speculations have been made (for more information, please refer to Cairns et al. [170]).

### 3.2. Amino Acid Metabolism

As previously introduced, glutamine metabolism is important for cancer cell survival and proliferation [84]. Therefore, overexpression of tumor glutaminase might not only decrease extracellular glutamine levels but could lead to high intratumoral glutamate levels. Briggs et al. showed that triple-negative breast cancer cells were able to secrete glutamate, leading to paracrine induction of HIF1α via inhibition of the xCT cystine/glutamate antiporter [171]. This report uncovered that the key oxygen sensor PHD2 (EglN1) controlling HIF1/2 stability is also capable to sense intracellular cysteine levels [171,172]. Furthermore, macrophages and DCs, which are often found in tumors and tumor draining lymph nodes, can also release glutamate in concentrations up to 30 µM [173]. Since T cells constitutively express the glutamate transporter mGlu5R, and that mGlu5R-induced adenylate cyclase can impede TCR-mediated T cell activation and IL-6 production, release of extracellular glutamate can therefore have a negative impact on T cell responsiveness [173,174]. A high concentration of extracellular glutamate can also affect other transporters, such as xCT cystine/glutamate antiporter. As aforementioned, xCT together with CD98/Slc3a2 form system x_c_^−^, which transports cystine (but not cysteine) into the cell in exchange for glutamate export. Consequently, high levels of extracellular glutamate might impair the import of cystine, possibly leading to ROS dysregulation and T cell dysfunction (as proposed by Siska et al. [175]). Glutamate receptors have also been found on other immune cells, including B lymphocytes and DCs [176], and future studies of the intratumoral glutamine/glutamate homeostasis may highlight new mechanisms of tumor-induced immune dysregulation.

### 3.3. Nucleotide Metabolism

Hypoxia can have different roles and especially that of allowing the increase of adenine nucleotide breakdown through the 5′ nucleotidase pathway, leading then to an accumulation of adenosine by tumor cells [177]. It has been shown that ATP is rapidly degraded to adenosine by the ectonucleotidases CD39 and CD73 expressed on tumor cells, which first convert ATP to AMP and then AMP to adenosine, respectively [178,179]. The accrued extracellular adenosine then binds to A2AR and A2BR (adenosine 2A and 2B receptors) expressed by T cells and NK cells, and induces intracellular cAMP accumulation and signaling, thus inhibiting both TCR-induced proliferation of T cells and IL-2 receptor expression [180,181]. In contrast, A2AR and A2BR blockade was shown to favor NK cell function by increasing Granzyme B expression and Perforin secretion, thereby promoting the anti-metastatic effects of NK cells [182,183]. Similarly, it was indicated that adenosine could enhance activation of anti-inflammatory M2 macrophages via A2AR and A2BR, inhibit TNF-α and release of IL-6 and IL-12, and augment IL-10 as well as VEGF production [184]. Notably, Young et al. recently showed a significant combination advantage in controlling in vivo tumor growth and lung metastases when A2AR and CD73 were both inhibited [185]. These encouraging observations led to the initiation of several clinical trials with small-molecule inhibitors targeting A2AR, but further explorations are still needed to prove the feasibility of such approaches in cancer patients [186].

## 4. Conclusions

In recent years, the field of cancer immunometabolism gained significant attention. Many of the recognized mechanisms of tumor immune escape appear to be selectively tailored for defined molecular immune pathways, as if tumor cells, through a “Darwinian evolutionary pressure”, were forced to lose/gain specific features in order to survive immune attack. Therefore, for the benefit of proliferating and expanding tumor tissues, a fine-tuned metabolic instrumentalization of the immune cells can occur in the microenvironment. For example, tumor acidity was envisaged as a sort of “protection armor”, by which cancer cells simultaneously abrogate the activity of all anti-tumor immune effectors and convert regulatory immune cells to pro-tumor allies. Another relevant example is tumor microenvironmental hypoxia, which dampens and neutralizes T cell functions and responsiveness. Thus, one may speculate that modulation of tumor microenvironment should contribute to a metabolic recovery of anti-tumor immune cells, and a relief of the detrimental effects exerted by immunosuppressive stroma components. Such an approach might be applied to improve spontaneous cancer immune control, or most likely to potentiate the efficacy of tumor immunotherapy. Recently, it has been shown that in tumor-bearing mice treated with checkpoint blockade therapy, such blockade increased the glucose concentrations in the extracellular tumor milieu and T lymphocytes from these mice displayed increased glucose uptake and glycolytic rates, augmented mTORC1 activity, and improved IFN-γ production [15]. Similarly, IDO inhibitors entered clinical trials and its inhibition when combined with checkpoint blockade therapy also showed promising results [187]. However, a recent Phase 3 study that combined the IDO inhibitor epacadostat with pembrolizumab, an anti-PD1 (programmed cell death 1) antibody, showed that adding epacadostat had no benefit [188]. Thus, since the number of mechanisms and possible targets is steadily increasing, a key question arises: can modulation of one metabolic pathway influence the outcome of immune cancer interaction to promote tumor regression? Although challenging, we speculate that future studies will aim to address the metabolic complexity of tumor microenvironment in its globality rather than target a specific gene or protein, especially through state-of-the-art technologies including transcriptomics analysis or high-throughput platforms testing compound libraries.

## Abbreviations

IDHs	Isocitrate dehydrogenases
D-2HG	D-2-hydroxyglutarate
α-KG	α-ketoglutarate
mTOR	Mammalian Target Of Rapamycin
CAR	Chimeric antigen receptor
OXPHOS	Oxidative phosphorylation
TCR	T cell receptor
FAO	Fatty acid β-oxidation
IFN-γ	Interferon gamma
GAPDH	Glyceraldehyde-3-phosphate dehydrogenase
AMPK	AMP-activated protein kinase
NFAT	Nuclear factor of activated T cells
ATP	Adenosine Triphosphate
FOXP3	Forkhead box protein P3
TCA	Tricarboxylic acid
NK	Natural Killer
Srebp	Sterol regulatory element-binding protein
ROS	Reactive oxygen species
NETs	Neutrophil extracellular traps
PPP	Pentose phosphate pathway
ASCT1/2	Amino acid transporter type 1/2
IDO	Indolamine-2,3-dioxygenase
GCN2	General control nonderepressible 2
tRNA	Transfer RNA
ASS1	Argininosuccinate 1
GSH	Tripeptide glutathione
APCs	Antigen presenting cells
HCC	Hepatocellular carcinoma
LmOVA	Listeria monocytogenes expressing OVA
IL	Interleukin
HIF1	Hypoxia-inducible factor 1
PDK1	Pyruvate dehydrogenase kinase 1
RORγt	RAR-related orphan receptor gamma
TAMs	Tumor-associated macrophages
VEGF	Vascular endothelial growth factor
REDD1	Regulated in development and DNA damage responses 1
MCT1/4	Monocarboxylate transporters 1/4
LDHA	Lactate dehydrogenase A
TLR	Toll-like receptor
DCs	Dendritic cells
MHC	Major histocompatibility complex
TNF-α	Tumor necrosis factor-α
GPCRs	G protein-coupled receptors
ICER	Inducible cyclic AMP early repressor
mAbs	Monoclonal antibodies
A2AR	Adenosine 2A receptor
A2BR	Adenosine 2B receptor
PD1	Programmed cell death 1
